# ‘One size does not fit all’: The value of person-centred analysis in health professions education research

**DOI:** 10.1007/s40037-020-00633-w

**Published:** 2020-12-07

**Authors:** Rashmi A. Kusurkar, Marianne Mak-van der Vossen, Joyce Kors, Jan-Willem Grijpma, Stéphanie M. E. van der Burgt, Andries S. Koster, Anne de la Croix

**Affiliations:** 1 Amsterdam UMC, Faculty of Medicine, Vrije Universiteit Amsterdam, Research in Education, Amsterdam, The Netherlands; 2 LEARN! Research Institute for Learning and Education, Faculty of Psychology and Education, Vrije Universiteit Amsterdam, Amsterdam, The Netherlands; 3 LEARN! Academy, Faculty of Behavioural and Movement Sciences, Vrije Universiteit Amsterdam, Amsterdam, The Netherlands; 4 Center for Evidence Based Education, Amsterdam UMC-location AMC, Amsterdam, The Netherlands; 5 Department of Pharmaceutical Sciences, Utrecht University, Utrecht, The Netherlands

**Keywords:** Person-centred analysis, Research method, Personalized approach

## Abstract

Health professions education (HPE) research is dominated by variable-centred analysis, which enables the exploration of relationships between different independent and dependent variables in a study. Although the results of such analysis are interesting, an effort to conduct a more person-centred analysis in HPE research can help us in generating a more nuanced interpretation of the data on the variables involved in teaching and learning. The added value of using person-centred analysis, next to variable-centred analysis, lies in what it can bring to the applications of the research findings in educational practice. Research findings of person-centred analysis can facilitate the development of more personalized learning or remediation pathways and customization of teaching and supervision efforts. Making the research findings more recognizable in practice can make it easier for teachers and supervisors to understand and deal with students. The aim of this article is to compare and contrast different methods that can be used for person-centred analysis and show the incremental value of such analysis in HPE research. We describe three methods for conducting person-centred analysis: cluster, latent class and Q‑sort analyses, along with their advantages and disadvantage with three concrete examples for each method from HPE research studies.

**Electronic supplementary material:**

The online version of this article (10.1007/s40037-020-00633-w) contains supplementary material, which is available to authorized users.

## Introduction

A quick scan of medical education journals shows that the research conducted in health professions education (HPE) predominantly employs what can be called variable-centred analysis [1]. This type of analysis, which investigates the relationships between two or more variables in a given sample, is important in understanding how variables in HPE research can influence one another. However, it can be hard for educators to adapt or change their practice on the basis of such analysis, as many studies focus only on a few variables and educational practice can be complex, context-dependent and messy. Person-centred analysis is an additional approach, which investigates how subgroups of individuals can be made based on how variables are related to each other across subgroups [1]. Person-centred analysis generates findings that could provide educators with tools to personalize practice initiatives. This article has threefold aims: to throw light on 1) how person/case-centred analysis can complement variable-centred analysis, 2) which methods can be used for person-centred analysis, and 3) how person-centred analysis can generate recommendations for more personalized support to students, faculty and everyone involved in HPE.

### What is person-centred analysis?

In a given dataset we can create groups of people in such a way that people with similar characteristics or similar scores on the independent variables are clustered together [2]. This is a type of case-based analysis, i.e. analysis of individuals or cases with similar characteristics. For this, we do not need to create a different type of file than what we would normally use for a variable-centred analysis. The only difference is the way the analysis is carried out. For some analysis types, special software may be necessary. If the associations with the dependent variables are computed by considering group membership as the independent variable, we demonstrate that group 1 with certain characteristics (e.g. high empathy and high resilience) shows a certain type of association with the dependent variables (academic performance), group 2 (e.g. low empathy and high resilience) shows a different or similar association with the dependent variables (academic performance), and so on. In person-centred analysis, the attempt is to find the ‘less obvious’ categories on the basis of patterns in the data. Statistically speaking we try to reduce the ‘noise’ in the data by splitting the total variability into ‘between-group’ variability and ‘within-group’ variability, and further concentrating on interpreting the differences between groups. The practical implications derived from these research findings can then be customized for the different groups as per their specific needs. Person-centred analysis complements variable-centred analysis, in which we look for associations between variables for the entire sample or subgroups in the sample made on the basis of demographic characteristics.

**Fig. 1:**
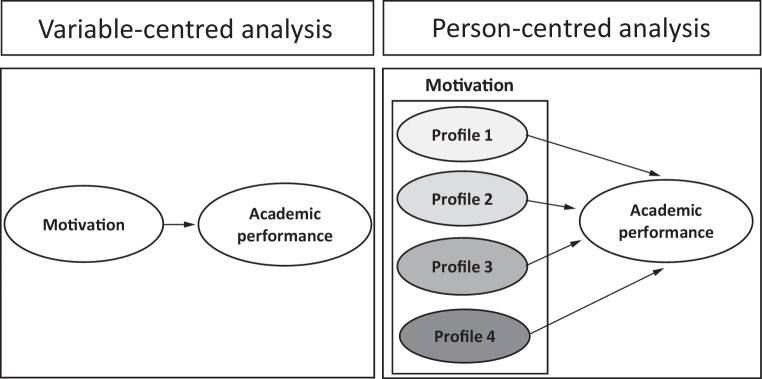
Depiction of an example comparing variable-centred [3] and person-centred analyses [2]

Person-centred analysis and its merits in understanding motivation and teaching-learning among school students has been described before by Vansteenkiste et al. in a study in which they computed motivational profiles of students based on a combination of their autonomous and controlled motivation [1]. See Fig. 1 for an example comparing variable-centred [3] and person-centred analyses [2]. In the current paper we take this work further by 1) considering the use of person-centred analysis for HPE research, 2) expanding this analysis beyond motivation research, and 3) adding more methods such as latent class analysis and Q‑sort analysis to the ones described by Vansteenkiste et al. (2009) [1].

Tab. 1 illustrates how variable and person-centred analyses for an example research question compare with as well as complement each other.

**Table 1:** Comparison of variable-centred and person-centred analyses with an example research question

	Variable-centred analysis	Person-centred analysis
*Research question*	How is empathy in HPE students associated with clinical performance?	How is empathy in HPE students associated with clinical performance?
*Method and analysis that can be used*	Collect scores on empathy using Jefferson’s Scale of Physician Empathy, collect clinical performance scores, run statistical analysis for association between the independent and dependent variables for the whole sample	Collect scores on empathy using Jefferson’s Scale of Physician Empathy, collect clinical performance scores, divide the sample into sub-groups of similar scoring students using a cluster analysis, run statistical analysis for association between subgroup membership as an independent variable and clinical performance as the dependent variable
*Implications of findings for educational practice*	Leads to general implications—If the association between empathy and clinical performance is positive, this can lead to an evidenced-based implication that training to foster empathy among students will help in better clinical performance (“One size fits all” approach)	Can lead to nuanced implications per subgroup—If the findings show that the groups with high and moderate empathy scores have good clinical performance and the low empathy score subgroup has poor performance scores, the implication would be that students with low, moderate and high empathy scores could receive different training programmes: modularization of education. (Personalized or “One size does not fit all” approach)
*Possible further research*	Provide empathy training to all students in the same manner and study the effects	Provide tailor-made empathy training to the students in each subgroup and study the effects of this personalized approach

## How to conduct person-centred analysis including concrete examples from the literature

In this section, we highlight three different methods that can be used for person-centred analysis: cluster analysis, latent class analysis and Q‑sort analysis. To exemplify this, we will present three studies for each of these methods. We chose these studies from the literature and our own experience, and analyzed and compared them in order to give a flavour of the practical implications person-centred analysis can provide researchers and educators with. In the Appendix of this paper, which can be found in the Electronic Supplementary Material, we have included details on these three methods, practical steps on how to conduct analyses using these methods, and how to interpret the findings of such analyses. We encourage the readers who are interested in more details to consult the online Appendix.

### Cluster analysis

Cluster analysis [4] is a method in which study participants are grouped together based on their scores or results on a combination of two or more variables. This method can be used with all kinds of sample sizes. It tries to reduce noise in the data by reducing ‘within-group’ variability and maximizing ‘between-group’ variability.

#### Jacobs et al. 2014 [5]

The aim of this study was to explore patterns among teachers’ conceptions for learning and teaching (COLT). The authors ran a cluster analysis using the participants’ scores on the three dimensions of the COLT: teacher-centredness, appreciation of active learning and orientation to professional practice. They accepted a cluster solution comprising five clusters. These five COLT profiles were labelled as *transmitters, organizers, intermediates, facilitators *and *conceptual change agents*. These profiles showed decreasing teacher centredness from transmitters to organizers. They then looked at how these profiles differed in their demographic characteristics and how they were distributed across two medical schools which differed in how long they were using a student-centred curriculum. Finally, they explained how faculty development activities could be tailored to suit these profiles, instead of using the principle of ‘one size fits all’.

#### Kusurkar et al. 2013 [2]

This study aimed to investigate the relationship between student motivation and performance. In this study, profiles of medical students from year 1‑6 were created on the basis of their scores on intrinsic and controlled motivation. Four profiles were found which were labelled as *high intrinsic low controlled, high intrinsic high controlled, low intrinsic high controlled *and* low intrinsic low controlled*. The associations of these profiles with learning and performance outcomes were then explored. Each of these profiles had different associations with these outcomes and the high intrinsic low controlled profile had the best outcomes in terms of more study hours, deep learning strategy, good academic performance and low exhaustion from study. In fact the high intrinsic low controlled profile only differed from the high intrinsic high controlled profile in its association with higher exhaustion from study, which was an important nuance in our findings. Recommendations were that these profiles would need different ways of monitoring and mentoring.

#### Orsini et al. 2018 [6]

The purpose of this study was to investigate dental students’ motivation and its academic outcomes. The authors created profiles of students on the basis of their intrinsic and controlled motivation. They found profiles similar to those of Kusurkar et al. 2013 [2], but different associations with study outcomes. They reported that high intrinsic motivation profiles, regardless of their controlled motivation, were associated with better perceptions of their own basic psychological needs, deep learning strategy, higher self-esteem and vitality, in comparison with the profiles with high controlled motivation. They also recommended a personalized approach towards interventions supporting students’ success and well-being.

More examples of this type of analysis are Sobral 2004 (medical students) [7], Tjin A Tsoi et al. 2015 (pharmacists) [8], and Van der Burgt et al. 2018 (medical specialists) [9].

### Latent class analysis

Latent class analysis [10] (LCA) is an exploratory, statistical technique that aims at forming subgroups (classes, clusters) of the samples included in a study, based on observed indicators of these samples. LCA can be used with categorical data. The output of LCA is a hypothesized grouping based on a combination of indicators.

#### Boscardin et al. 2012 [11]

This study aimed to identify students for remediation and to contribute to consensus about the best methodological approach for remediation. LCA was used to analyze scores of 147 medical students on the Clinical Performance Examination. Three distinct performance profiles were identified, including two low performing subgroups. Distinguishing two different low performing subgroups had significant implications, as the two groups had low scores on contrasting sets of performance indicators. The first subgroup of students showed deficits in both clinical knowledge and all kinds of clinical skills, while the second subgroup mainly displayed a deficit in communication skills. The results of this person-oriented study offer educators the possibility to target the remediation needs of low performing students with more specificity based on their individual performance profile compared with a variable-centred analysis.

#### Mak-van der Vossen et al. 2016 [12]

The purpose of this study was to identify patterns in the behaviours of medical students who received an unsatisfactory professional behaviour evaluation in medical school, and to define a variable that could be used for the categorization of these patterns. A latent class model with various choices for the number of latent groups was fitted to the response data. In this case, the response data indicated whether each of 109 unprofessional behaviours, as earlier summarized in a template based on a literature review, was described as ‘*observed’* or ‘*not observed’* in student evaluation reports. LCA yielded three classes of students who received unsatisfactory professional behaviour reports: *‘poor reliability’, ‘poor reliability and poor insight’*, and *‘poor reliability, poor insight and poor adaptability’*. Based on the content of the three classes, the latent variable was described as ‘capacity for self-improvement and adaptability.’ Once the classes were finalized, the categorical data of representatives or ‘prototypes’ (the top 10 students with the highest probability to belong to that class) were put together to provide each class with a narrative profile description. These profiles showed differences in the recurrence and intensity of unprofessional behaviour. This study suggested that each of these unprofessional behaviour profiles needs to be handled differently depending on the capacity for self-improvement and adaptability of the students.

#### Lambe & Bristow 2011 [13]

The focus of this study was to identify a model of ‘typologies’ of student performance. LCA was used to make subgroups of students based on measures of prior academic achievement, interview rating at the time of medical school admission and outcome measures of subsequent performance across the course. LCA identified a three class model of distinct subgroups representing ‘typologies’ of student examination performance. This study illustrates that LCA has the potential to inform the selection process; three different patterns in indicators of students who are most likely to need learning support were identified. This support can be personally customized based on these typologies generated by LCA.

Other examples of studies in HPE in which LCA has been applied are Regehr (2012) [14], who created different typologies based on residents’ performance assessments, and Gingerich (2014) [15], who created typologies of physicians social judgement of residents’ performance assessment.

### Q-sort analysis

Q‑methodology is suitable for the study of subjectivity (e.g. viewpoints, ideas and opinions) [16–18]. It uses stimuli (usually in the form of statements) that participants need to rank order according to agreement. A special form of factor analysis is used to group participants who think similarly about the topic under study.

#### Fokkema et al. 2014 [19]

This study aimed to determine the perceptions of obstetrics-gynaecology residents and attending physicians about workplace-based assessment. There were 36 statements and 65 participants. The authors found five types of perceptions: *enthusiasm, compliance, effort, neutrality*, and *scepticism*. The issues underlying these five profiles were ideas about intended goals of the innovation, its applicability, and actual impact. The authors used their findings to support the design and implementation of workplace based assessment. They felt that the study ‘may help colleagues understand one another’s responses to an innovation’. The generated profiles need to be supported in different ways to get their whole-hearted participation in order to make the workplace based assessment innovation successful.

#### Dotters-Katz et al. 2016 [20]

This study focused on US medical graduates’ attitudes and motivation for teaching. Forty-seven statements were used. Through convenience sampling, 107 residents ‘from a wide variety of specialties and postgraduate year levels’ joined the study, and the Q‑sorting and post-interview were done digitally. Their analysis yielded three profiles*: enthusiasm, reluctance *and *rewarded.* These findings were used to inform modifications in the design of resident-as-teacher programmes that ‘reinforce and encourage attitudes that promote teaching as well as improve trainees’ motivation to teach’, for example by making an effort to improve the motivation of reluctant teachers and encouraging positive attitudes among rewarded teachers.

#### Berkhout et al. 2017 [21]

Berkhout and colleagues aimed to find patterns in students’ self-regulated learning behaviours in the clinical environment. They created 52 statements from theory and student interviews. Four students in 11 different clinical clerkships, in different hospitals, sorted the statements. An online data collection procedure was used and there was no ‘live’ post-sorting interview. Their analysis led to five patterns of self-regulated learning behaviour, which they called *engaged, critically opportunistic, uncertain, restrained *and* effortful*. The authors recommended a more ‘individualized approach’ to supervision and support taking into account the differences between student profiles.

## Comparisons, advantages and disadvantages of the three methods for person-centred analysis

This paper sets out to sketch a person-centred analysis in HPE research, its advantages and implications for practice. The person-centred analysis can be used to study concepts and design tailor-made interventions for almost all topics in HPE, such as teaching-learning methods, student and teacher motivation, professionalism, academic and clinical performance, assessment, faculty development, and leadership training. The overall implications of person-centred analysis using concrete examples mentioned in the earlier section are summarized in Tab. 2.

**Table 2:** Implications generated from the use of person-centred analysis in the included studies

Cluster analysis	Latent class analysis	Q‑sort analysis
*Jacobs et al. 2014* [5]	*Boscardin et al. 2012* [11]	*Fokkema et al. 2014* [19]
Generated 5 profiles of teachers on the basis of their conceptions of learning and teaching, which had implications in the form of personalized faculty development activities	Generated 3 profiles based on students’ clinical performance which were used to customize remediation activities for improvement of performance	Generated 5 profiles on the basis of residents’ and physicians’ perceptions of workplace based assessments and made personalized recommendations for introduction of innovations in workplace based assessments
*Kusurkar et al. 2013* [2]	*Mak-van der Vossen et al. 2016* [12]	*Dotters-Katz et al. 2016* [20]
Generated 4 profiles of students on the basis of the combination of their intrinsic and controlled motivation which had implications for academic performance, exhaustion from study and learning approaches	Generated 3 profiles of unprofessional behaviours of undergraduate students which gave an insight into what customized remediation measures could be used for each profile	Generated 3 medical graduate profiles on the basis of their attitude and motivation to teach in order to customize faculty development activities
*Orsini et al. 2018* [6]	*Lambe and Bristow 2011* [13]	*Berkhout et al. 2017* [21]
Generated 4 profiles of students on the basis of the combination of their intrinsic and controlled motivation which had implications for study success and well-being	Generated 3 student profiles based on prior academic achievement and interview rating at the time of medical school admission to identify students who are most likely to need learning support	Generated 5 student profiles on self-regulation of their clinical learning in order to personalize support and supervision

The specific advantages and disadvantages of the three analysis methods are compared and summarized in Tab. 3.

**Table 3:** Advantages and disadvantages of the methods for person-centred analysis

	Cluster analysis	Latent Class analysis	Q‑sort analysis
Type of data	Can be used for continuous or categorical data	Can be used for continuous or categorical data	Can be used for a combination of rank-ordered statements and interview data
Required sample size	A good sample size is important for cluster stability. A thumb rule is a sample size of minimum 100	Medium size (at least 70 samples) as well as (very) large sample sizes can be handled, depending of the number of indicators in a given sample	Sample size can be relatively small (65 participants [18]) or larger (152 participants [21]). The quality of the sample is more important than the quantity of the sample. Researchers try to select a varied and diverse sample
Advantages	– Provides more generalizable findings owing to the nature of the data and the ability to handle large sample sizes – Can lend itself to longitudinal follow-up of profiles to see if they change over time	– Flexibility of the model specification in LCA provides advantage over cluster analysis, which may not yield an optimal representation of the profiles [19] – Can lend itself to longitudinal follow-up of profiles to see if they change over time – Statistical and interpretational criteria are used to determine the optimum number of clusters, which means that researchers themselves can determine the number of classes and determine an understandable and practical to use ‘latent factor’ that describes the difference between classes	– This is a robust and systematic way of studying subjectivity. It can be precise and rigorous (depending on the choices made while conducting the analysis), and yet keeps the richness of descriptive data by including post Q‑sort questions or interviews
Disadvantages	– Because of its exploratory nature, it may be random and not generate similar profiles in different samples – Cannot be used for small sample sizes	– Limited generalizability – Sample size should not be too small, especially if there is a large number of indicators. This problem might be solved by using a penalty parameter while statistically estimating the latent class model	– Limited generalizability. Q‑studies are not designed for generalizability purposes, they are for uncovering authentic viewpoints within the sample (and sample should be high quality). From there, if desired, prevalence of viewpoints can be tested in larger population through other methods – Difficult to study change in profiles over time

Please refer to Kusurkar et al. as an example of a paper which employs person-centred analysis and uses this analysis to give concrete recommendations for practice for the generated profiles [22].

### Limitations and ethical considerations of person-centred analysis

Subgroups found in samples may be culturally sensitive and context-dependent. The profiles and findings from this analysis could thus be difficult to generalize to other populations. To use the results of person-centred analyses for designing practical interventions, it is better to investigate the profile structure in the local target population. A person-centred analysis is not a replacement for variable-centred analysis, but a complementary analysis. A risk of person-centred analysis is that it can lead to stigmatization of certain groups. To minimize this risk, it is important that:

A. Researchers using person-centred analysis always do the following while applying for ethical approval and publishing their research:
  Explain the background and rationale for conducting such an analysis;
  Explain how the results of this analysis should be interpreted, especially keeping in mind its context; and
  Make a declaration that the results of such research should be used in a constructive way to customize interventions and not to stigmatize certain groups.
B. Ethical Review Boards always consider the following:
  Have the researchers provided a good rationale for using person-centred analysis?
  Are the researchers actually using the generated profiles for tailor-made or personalized interventions?
  How have the researchers clarified how they will treat the findings from this analysis?

## Conclusion

Person-centred analysis using cluster, latent class or Q‑sort methods can complement the currently predominant variable-centred analysis in HPE research for a variety of topics. This analysis provides distinct advantages for developing a personalized approach towards teaching, supervision, assessment and faculty development activities, as illustrated by the examples included in this paper.

## Caption Electronic Supplementary Material

Appendix. Methodological details of cluster, latent class and Q‑sort analyses
