# Supplementary material for: ‘One size does not fit all’: The value of person-centred analysis in health professions education research
Source: Perspect Med Educ. 2020 Dec 7;10(4):245–51. doi: 10.1007/s40037-020-00633-w (PMC8368141; doi:10.1007/s40037-020-00633-w)
Supplement: Supplementary file 1 — Appendix. Methodological details of cluster, latent class and Q‑sort analyses [file 40037_2020_633_MOESM1_ESM.docx]

**Appendix**

Kusurkar, R.A., Mak-van der Vossen, M., Kors, J. *et al.* ‘One size does not fit all’: The value of person-centred analysis in health professions education research. *Perspect Med Educ* (2020). <https://doi.org/10.1007/s40037-020-00633-w>

**Methodological details of cluster, latent class and Q-sort analyses**

**Cluster analysis**^1^ - This analysis can be done quite easily using SPSS. Two ways of conducting this analysis are K-means clustering and hierarchical clustering. In SPSS, an additional ‘Two Step’ clustering procedure can be used to suggest an optimal cluster number.

K-means clustering is the most commonly used data clustering method. The methods sorts cases in a predefined number of clusters. The number of clusters can be based on theoretical (existing literature) or practical (applicability) considerations. Initial k-cluster centers are selected and then iteratively refined assigning each data point to its closest cluster-center and updating each cluster-center to be the mean of its constituent data points. An acceptable cluster solution should explain at least 50% of the variance in the variable scores and have an incremental effect over the cluster solution with (k-1) groups.

Hierarchical clustering is an approach in which all data points are clustered hierarchically until only one cluster is left. The optimal cluster solution is decided on the basis of a hierarchical diagram called a dendrogram, a taxonomy or hierarchy of data point. This is a convenient representation which answers questions such as: ‘How many useful groups are present in this data?’ and ‘What salient interrelationships are present?’.^2^

Hierarchical clustering techniques are fundamentally different from K-means clustering. K-means tries to find compact clusters, where cluster members are similar (as far as possible). Hierarchical clustering leads to a tree of clustering, where it remains arbitrary at what level you want to set the borders between clusters.

After using one of the cluster methods, the cluster solutions can be tested for stability using a double split cross validation procedure in which the sample is divided into two and the cluster solution with the same cluster centers is tested in each sample. For a stable cluster solution, the Cohen’s kappa values, derived from this procedure, should be as close to 1 as possible.^3^

**Practical steps for K-means cluster analysis:**

- Prepare your data file in SPSS just like for any other analysis.
- Compute standardized scores (z-scores) for the variable which you would like to use to make the clusters.
- Exclude outliers from the data as cluster analysis is sensitive to outliers.
- Use the command “Classify” and enter the number of clusters (“n”)that you would like to test (start with 2 and then go on with 3 and more), choose “save assigned cluster”.
- Then repeat the process with “n+1”, “n+2”, “n+3” clusters.
- Check for the percentage of variance explained by the 2-cluster, 3-cluster, 4-cluster, etc. solutions. Using a benchmark of at least 50% variance explained, choose the cluster that explains a significant amount of variance The optimal number of clusters can be selected on the basis of statistical parameters and interpretability.
- Once you have chosen a cluster solution, create two new files splitting your sample into two random subsamples. Run the clustering analysis on each subsample and see if you get similar clusters in both. Compute the Cohen’s kappa for checking the stability of the cluster solution.
- Use cluster membership as the independent variable and run t-tests or Analysis of Variance or Multiple Analysis of Variance for the dependent variables of interests to see the relationship of the different clusters with the outcome measures.

**Interpretation of findings:**

- Try to understand the meaning of the clusters based on your hypothesis, theoretical framework and the scores on the variables used for clustering.
- If possible label the clusters (without being judgemental) and provide a description of each cluster so that your interpretation becomes clear to the readers or practitioners.
- Try to understand how the cluster characteristics are associated with outcome variables.
- Before ascribing any meaning to the clusters, it is important to establish the cluster stability mentioned above.
- Be cautious in projecting your findings to other contexts and cultures.

For use on categorical data, this data needs to be treated first (e.g. with Homogeneity analysis using alternating least squares - HOMALS).^4^

**Latent Class Analysis**^5^ – This is also called Latent Partition Analysis (LPA). This is done in a manner that the samples in the study are homogeneous within, and heterogeneous between the formed subgroups. It is a flexible method, as the best fitting model is established by testing several combinations of numbers of classes. This can be done using the software programmes R^6^ or Latent Gold^7^.

LCA can be used if there exists a still-unknown, so called ‘latent’ variable that can be used to make subgroups of the samples under investigation. This newly emerging variable can be identified as a distinguishing factor for the content of the subgroups. The researchers then determine if the distinguishing factor has practical relevance, and attribute a meaningful description and name to it.

LCA has an advantage over other clustering methods because it can reveal patterns, i.e. combinations of indicators within a sample, that cannot easily be detected by other methods. LCA is a probabilistic method. It means that there is no one-to-one relationship between a class and the occurrence of an indicator within that class, but each class is composed of a subgroup that is more likely to display a certain pattern than the subgroup belonging to a different class. A similar classification process is applied in diagnosing a disease: The presence or absence of a certain symptom in a patient (indicator in a sample) does not always lead to one specific diagnosis (class), but a certain combination of symptoms (pattern) makes this diagnosis more likely. Thus, instead of making a black-and-white decision on the subgroups of samples as cluster analysis does, LCA defines the probability of certain patterns in the samples, and thus sketches a more attenuated picture.

LCA has the possibility of defining ‘prototypes’ in each subgroup. To achieve this, LCA specifies for each class a probability of a sample belonging to that class. The probabilistic statement indicates the certainty of the assignment of a sample, based on a certain combination of indicators, to that class. In particular, samples that have a high, say >90%, probability of belonging to a certain class could be considered as prototypes of that class.

**Practical steps for Latent Class Analysis:**

- Conduct thematic or content analysis of your descriptive data.
- Convert the categorical data into binary response data, e.g. presence/absence of the indicator in each sample (SSPS or Excel file).
- Put your binary data into one of the abovementioned software programmes.
- Test different numbers and properties of classes.
- Determine the best fit for the number and properties of classes by considering the following:
  - the statistical information indicating between class differences and within class homogeneity.
  - the practical relevance of the content of the classes.
  - the number of cases per class.
- Define prototypes for each class by taking the samples that have the highest probability to belong to that class (e.g. the top 10).
- Provide the prototypes of each class with narrative information from your descriptive data to generate profile descriptions for each class.

**Interpretation of findings:**

- Try to understand the meaning of the classes based on the practical relevance of the content of the classes and the descriptions of the prototypes.
- Identify the latent variable that distinguishes the classes from each other, and give this variable a meaningful name.
- Be aware that the samples are clustered into *hypothetical* patterns (the classes) based on the *chance* that they display a combination of indicators.
- Be cautious in projecting your findings to other contexts and cultures.

**Q-sort analysis**^8-10^- Although there is considerable flexibility in Q-methodology, there are some common practices. A Q-methodological study starts with the development of a set of statements on a topic (the Q-set). This set of statements can be created as a result of interviewing stakeholders, looking at teaching evaluations, theories and literature, focus groups, etc. This initial Q-set is often piloted and refined before use in a study.

Each participant sorts statements in a Grid (the Q-sort), with most statements placed in the middle, and the fewest placed at the edges (i.e. bell-curve shaped). These edges have 'strongly agree' or 'very important' on one side, and 'strongly disagree' or 'not at all important' on the other. This ranking process is called ‘Q-sorting’ and forces participants to make choices based on their own opinions and experiences. Usually the Q-sorting procedure is followed by a post-sorting interview or survey questions. In this post-sorting (often semi-structured) interview, or in some open survey questions, the participant elaborates on the reasons and stories behind the Q-sort, to enrich the data collected from the Q-sort.

Q-sorts are then compared to identify groups of individuals (profiles) who have similar attitudes on the subject of interest. This is often done using using Q-sort analysis software called PQmethod.^11^ The ranking scores are analysed statistically to lead to different factors^10^ using Q-sort analysis software. The number of profiles are dependent on how the participant scores 'load' onto a specific profile, similar to factor analysis. The qualitative data can aid the decision for the number of factors/profiles. The profiles are finalized through a combination of statistical, methodological and qualitative data analysis from a post Q-sort interview or survey questions. A description of the prototype of each profile is constructed by the researchers while constantly consulting the data.

**Practical steps for Q-sort analysis:**

- Develop a set of statements from the literature and pilot them with some study participants, refine them and your Q-set will be ready.
- Select participants using theoretical sampling strategies, in order to include participants with diverse viewpoints.
- Ask participants to sort the statements into the Q-grid, and ask participants to elaborate on their choices.
- Enter the Q-sort of each participant into the abovementioned software and run the Q-sort analysis.
- Check the different solutions for predetermined statistical criteria. At the minimal , you should take into account the percentage of variance explained by different solutions, eigenvalues, and number of Q-sorts per factor, total number of Q-sorts loading significantly on one factor, and Q-sorts loading on more than one factor or no factor at all.
- Check the different solutions for methodological criteria: are the factors coherent, differentiated and recognizable?
- Check if the qualitative data (from post-sorting interview) corroborates the factor solution.

**Interpretation of findings:**

- Try to understand the meaning of the profiles based on your research question and theoretical framework.
- Combine the result from the factor analysis with the answers the post-sorting questions to create a rich and accurate profile description.
- Label the profiles to capture their essence and improve the reader’s capability of comparing and contrasting the findings.

**References**

1. Antonenko PD, Toy S, Niederhauser DS. Using cluster analysis for data mining in educational technology research. Educ Tech Res Dev. 2012;60:383-98.
2. Murtagh F, Contreras P. Algorithms for hierarchical clustering: an overview. Data Mining Knowl Discov. 2012;2:86-97.
3. Kusurkar RA, Croiset G, Galindo-Garré F, Ten Cate TJ. Motivational profiles of medical students: Association with study effort, academic performance and exhaustion. BMC Med Educ. 2013;13:87.
4. De Wolff MS, Van IJzendoorn MF. Sensitivity and attachment: A meta-analysis on parental antecedents of infant attachment. In: Annual progress in child psychiatry and child development. 1998. Eds. Hertzig ME, Farber EA. ©1998, Philadelphia, USA: Taylor & Francis Publications. pp. 25-56.
5. Vermunt JK, Magidson J. Latent class cluster analysis. In: Applied Latent Class Analysis. Eds. J Hagenaars, A McCutcheon. Cambridge: Cambridge University Press. 2002; pp: 89-106.
6. Team RC. R: A language and environment for statistical computing. R Foundation for Statistical Computing, Vienna, Austria, 2012.
7. Vermunt JK, Magidson J. Technical guide for Latent GOLD 4.0: Basic and advanced. Belmont, MA: Statistical Innovations. 2005.
8. Brown SR. 1980. Political Subjectivity: Applications of Q Methodology in Political Science. New Haven, CT: Yale University Press.
9. Stephenson W. Introduction to Q-Methodology. Operant Subjectivity. 1993;17:1-13.
10. Watts S, Stenner P. Doing Q Methodological Research: Theory, Method and Interpretation. Los Angeles, CA: Sage Publications. 2012.
11. Schmolck, P. PQmethod (Version 2.35). 2014. Retrieved from <http://schmolck.org/qmethod/>
